# A preliminary exploration of experiences of integrating the body in the self in two women with anorexia nervosa in view of phenomenological conceptualisations

**DOI:** 10.1186/s40337-022-00675-x

**Published:** 2022-11-01

**Authors:** Caroline R. Naess, Liv-Jorunn Kolnes

**Affiliations:** 1 Bergen, Norway; 2 VID Specialized university, Diakonveien 12-18, 0370 Oslo, Norway

**Keywords:** Anorexia nervosa, Phenomenology, Embodiment, Norwegian psychomotor physiotherapy, Therapeutic relationship, Bodily connectedness, Articulating feelings

## Abstract

**Background:**

People with anorexia nervosa often present with confusions around bodily sensations and difficulties in experiencing their body as a place of their own. Many existing models understand anorexia nervosa as a disorder of behaviour and thoughts about eating and body size, and treatments typically focus on modifying thoughts and psychological processes. They leave aside the subject as she experiences the body from a first-person perspective. Inspired by phenomenology and the embodied mind thesis, this explorative study examines in depth the experiences of individuals with anorexia nervosa as they engage in Norwegian psychomotor physiotherapy. Through release of bodily tension and restricted breathing, this method aims to help subjects tune into the body and accept that difficult experiences, relationships and feelings are also bodily.

**Methods:**

Qualitative in-depth interviews were conducted with two women who had been attending Norwegian psychomotor physiotherapy for more than two years. Thematic analysis was used to identify, analyse and interpret themes within the data.

**Results:**

Three main overarching themes that structure the subjects’ experiences were identified: the meaning of the therapeutic relationship, changes in bodily connectedness and ways of moving, and improved ability to articulate and reflect on feelings.

**Conclusion:**

The subjects described a greater tendency to experience the body in the self and as a place of their own, a more flexible and vital body, and an increased capacity to identify, express and make sense of feelings. These changes enriched their interactions with the social world. Feeling acknowledged and accepted by the therapist throughout the process was essential. The study illustrates how difficult experiences, thoughts and feelings can, over time, manifest in the body as withheld breathing and diverse bodily constraints affecting both self- and body awareness. The study endorses the phenomenological concepts that our experiences of the self and the world are essentially bodily, and emphasizes the importance of the first-person perspective when investigating the contribution of the body to the self and to our interactions with the social world. Physio- and body awareness therapies that help patients relate to, understand and integrate bodily experiences may complement other treatment approaches and help patients with anorexia proceed with the recovery process.

**Plain English summary:**

Having anorexia nervosa involve changes in the way one experiences the body. The condition has been linked to confusions around bodily sensations and reduced experience of the body as an integrated place of their own. The purpose of this study was to gain a richer understanding of bodily experiences in subjects with anorexia nervosa having attended a specialized physiotherapy approach. In depth interviews were conducted with two women who had anorexia nervosa and who had engaged in Norwegian Psychomotor Physiotherapy for more than two years. Three themes that structure the subjects’ experiences were identified: the meaning of the therapeutic relationship, changes in bodily connectedness and ways of moving, and improved ability to articulate and reflect on feelings. The subjects described a greater tendency to experience the body in the self and as a place of their own, a more flexible and vital body, and an increased capacity to identify, express and make sense of feelings. Feeling acknowledged and accepted by the therapist throughout the process was essential. The study illustrates how difficult experiences, thoughts and feelings can manifest in the body as withheld breathing and diverse bodily constraints affecting both self- and body awareness in people with anorexia nervosa.

## Background

Anorexia nervosa (AN) is an eating disorder characterized by a refusal to maintain minimal normal body weight, an intense fear of weight gain, denial of the seriousness of the current low weight and disturbances in perceptions, attitudes and beliefs pertaining to one´s body (i.e., body image) [1]. It is associated with considerable physical and psychological morbidity, including medical concerns related to severe underweight, anxiety, depression, obsessive-compulsiveness, and post-traumatic stress. AN has also been linked to a tendency to supress negative feelings and thoughts as affected individuals seek to avoid conflict with others [2]. The illness affects both men and women, although incidence is substantially higher in women. Onset typically occurs during the teenage or young adult years, with prevalence rates highest in females between the ages of 15 and 25 [3]. As the nature of the disorder is not yet fully resolved and in view of the lack of agreement regarding effective treatment approaches, AN is associated with poor prognosis and relapse rates are high [4, 5].

Many questions remain about how to construct optimal treatments for individuals with AN. In addition to restoration of a healthy body weight, many existing models use psychological theory to address factors in clinical treatment and include interventions focusing on behaviour, cognition and psychological processes [6]. Treatment for body image disturbances focuses on affective, cognitive, and behavioural aspects of the disorder, and typically addresses features such as size perception and negative and unrealistic thoughts and feelings about the body (e.g., overestimating body size or the size of a particular part of the body), as well as anxiety-provoking situations, body checking and reassurance seeking [7–9]. In short, these models understand AN as a disorder of behaviour and thoughts about eating and body size, and treatment focuses on changing eating behaviour, attitudes and cognitions related to body image.

And yet, having a psychiatric disorder tends to involve a transformation of one´s life on multiple levels. It involves changes to one´s sense of self, one´s body, and it changes one´s way of being in and relating to the world [10]. The bodily being in the world and the body as the centre of experience is in particular disrupted. Habits, expectations, abilities, and meaning structures may become distorted, and the fundamental sense of one´s body and the embodied normalcy in which habits and values are rooted, may be interrupted [10]. Such transformations may have a particular tendency to materialise in subjects with AN, considering that the condition has been linked to confusions around bodily sensations and difficulties associated with sense of self and embodiment [11]. A reduced capacity to perceive and recognize bodily sensations and signals is acknowledged in this population [12–14], and diverse constraints in the musculoskeletal system affecting posture, breathing and movement have been identified in women with AN [15, 16]. Furthermore, it has been contended that people with AN often lack the experience of the body as an integrated place of one´s own and that they suffer from a tension between consciousness of both its physicality and its subjectivity, which in turn influences their bodily self-awareness [17]. This tension between subjectivity and physicality may trap the subject in a vicious cycle preventing her from integrating the body in the self and from restoring health. Legrand claims that individuals with AN have a tendency to treat their body as a closed system, so that the body encloses the self, shutting out the outside world [17]. Yet, if the outside is shut out, the relational and social world will inevitably become restricted.

Accordingly, given the essential role of the body in the experience of the world and self, it may not be sufficient to merely understand and manage AN as an essentially psychological disorder characterized by behavioural and cognitive disturbances. One problem with treatment models primarily addressing thoughts and psychological processes is that they leave aside the subject as he/she experiences the body. They do not capture, in Zahavi´s words, “the body as it is lived through from an embodied first-person perspective” ([18], p 7). How the sense of self and interactions with others are mediated by the body is equally often unaccounted for.

### Theoretical context: approaching anorexia nervosa as an embodied experience

To help us achieve a richer understanding of the existential transformation anorexia brings about in the subject, phenomenology and the embodied mind approach may provide fruitful theoretical conceptualisations from which to examine the body in those affected. Phenomenology is concerned with revealing and describing structures and conditions of conscious and embodied experience by taking the first-person perspective of lived experience as the starting point for its investigations [19]. Phenomenology of the lived body and embodiment theories have become major conceptual frameworks for understanding the mind in general and the domain of mental illnesses in particular during the past decades [11, 20–25]. Within the cognitive science literature it is widely argued that to understand the mind, its fundamental situated character must be taken seriously, and that higher level processing is grounded in the organism´s sensory and motor experiences [23]. The embodiment thesis infers that “the subject is constitutively bodily” ([20] p. 208) and that “mental activity depends essentially not just on the brain but on the body as well” ([26] p. 279). For the self to belong to the world, “there is no other way than being corporeal. Not only being an experiencing subject, but more specifically being an experiencing body is necessary for there to be an experienced world at all” ([20] p. 209). In this view, investigations of behaviour and cognition should involve considerations of the contribution of the body to our self and to our interactions with the world.

The dominant view among many neuroscientists and philosophers has long been to associate consciousness predominantly with the brain. This brain-bound approach implies that the brain directly determines what you experience, and that the body is inessential for conscious experience [27]. Since the mid 1990s, scholars have proposed a different way of thinking about the mind and the brain–body relationship. Influenced by thinkers from phenomenology (e.g., Edmund Husserl, Maurice Merleau-Ponty, Jean-Paul Sartre), they claim that a brain-bound approach is not sufficient to account for the nature of the mind. To fully account for our mind and our mental life, we also need to consider the body. An embodied mind view, indicating that the biological requirements for consciousness include a living body and its sensory-motor experience interacting with its environment, and not just neuronal processes in the brain, was put forward [28]. In this approach, body and mind form a genuine experiential unity by which “the psychic obtains its position in space and time” ([29], p 176). The body is fundamental in our experience and perception of the self and the surrounding world. It is a position of distinctive sensations that can only be felt firsthand by the embodied experiencer concerned [30]. Yet, the body is not an extended physical substance to the soul, it is not about a unification of two distinct heterogeneous constituents, as the unity of the body and soul is unique in that the two are not only inseparable but also indistinguishable ([31], p 46).

Merleau-Ponty [32] differentiated carefully between the perceived body (i.e., body image) and the dynamic sensory-motor experience of the body in its environment (i.e., the lived body). *Body image* has been defined as a “picture of our own body which we form in our own mind” ([33], p 11). Over time, the concept has been linked to mental representations, perceptions, attitudes, beliefs, and dispositions pertaining to one´s body that can be captured in an objective way [34, 35]. *The lived body* has been described as a lived centre of experience [32]. It is the body as we experience and sense it from a first-person perspective, it is about how we feel towards and perceive others and the world in which we live, and it enables us to relate ourselves to other bodies and to other minds [32]. Although the claim runs counter to traditional Western philosophy centred on rationalism and the concept of a disembodied mind, Merleau-Ponty contended that attempts to understand human nature should account for the lived body and perception as the bases of subjectivity [32]. For Merleau-Ponty, perception is itself an embodied activity in which the body is the condition of possibility for perception and action. While the body on one hand is a physical object that can be weighed, measured and described in physical or naturalistic terms, the body is also the source of subjective feelings, perceptions and sensations; it plays a critical role in the constitution of subjectivity and is where consciousness takes place [32]. Given the inseparability of embodiment, perception, action and subjectivity, changes to one´s body may thus lead to changes in one´s sense of self and in one´s way of being in the world ([10], p 27).

To better understand and manage AN, and to achieve a richer understanding of the role of the body in the self and in experiencing and sensing the relational and social world, it may be helpful to expand the focus from viewing the illness as an essentially behavioural and cognitive disturbance to looking at AN from a phenomenological point of view. Drawing on the phenomenological concept of embodiment, implying that the body has a crucial role in the experience of the self and of social encounters and focusing on the lived body as it is experienced from a first-person perspective, investigations of living with AN may benefit from involving the subjects being in the world brought forth by their subjective and intersubjective experiences [28].

Given the challenges associated with treating AN, and the fact that the condition has been linked to confusions around bodily sensations and to difficulties in integrating the experience of the body in the self, the aim of this study is to explore in depth experiences concerning the body in subjects with AN who have been attending Norwegian psychomotor physiotherapy (NPMP) sessions and to summarise what the interviewees consider to be helpful in such a treatment approach. The main characteristics of NPMP are detailed after the Methods section.

### Research addressing bodily connectedness in individuals with anorexia nervosa

Aside from two papers exploring clinical experiences of applying NPMP principles to individuals with AN, research describing NPMP from the perspective of the subject is limited. The two aforementioned papers discuss the potential of NPMP-based therapies in terms of increasing body contact, initiating novel ways of sensing and interpreting bodily signals, and improving emotional awareness through the body [15, 36]. They report that patients, whose experiences were put into words during verbal and/or written reflections following individual or group therapy sessions, experienced bodily signals differently, were able to articulate feelings more adequately, felt more connected to the body and experienced enhanced ownership of the body after attending weekly physiotherapy and/or body awareness group therapy [15, 36]. Similarly, a third paper based on the journal entries of a patient with AN suffering from muscular tensions and pain, reported new bodily sensations and movement patterns emerging during an NPMP treatment course [37]. In addition, it was assumed that the treatment helped support the bearing structures of the self and helped the patient understand experiences from the past and make sense of those in the present [37].

A small number of studies exist in which the aim was to enhance body awareness (often referred to as ‘body awareness therapies’) and to apply body-oriented interventions for subjects with AN. The focus of these treatments overlaps to some degree with the aims of NPMP, such as increasing postural stability and flexibility in movements, reducing muscular tension, and achieving free breathing. Having reviewed body awareness interventions (including massage therapies and exercises with the aim of changing breathing, posture and the ability to relax) for subjects with AN, Probst, Vancampfort and colleagues concluded that such interventions provide positive effects on bodily awareness and satisfaction, weight restoration, eating pathology, and quality of life [38, 39]. While interventions involving muscular and aerobic rehabilitation [40, 41] are not specifically directed at increasing body awareness, they may implicitly contribute to improved bodily connectedness, as increased general muscle strength may facilitate the experience of a strong and stable body that feels more centred and of which one is in control [16].

Another study – based on a hospitalized patient – focused on adapting interventions to the patient’s physical and psychological potential in close collaboration with the multidisciplinary team at the unit, including the specialist physician, nursing and dietary staff [42]. Along with medical stabilization, the main focus of the physical therapy was to promote functional independence in daily living activities through the restoration of the body (e.g., stretching short muscles, gait training, resistance training, endurance and postural stability training) [42].

In general, individual physical therapy interventions are based on taking a history and conducting a body examination, thereby enabling the intervention to be adapted to the specific needs of the subject. With the exception of Fisher and Schenkman [42], previous studies presenting physical therapy and body awareness interventions for subjects with AN do not describe results from the body examinations. Indeed, a thorough bodily assessment is crucial in establishing the functionality of the body and specific bodily concerns (e.g., postural dealignments, constrained breathing, and muscular tension) on which the interventions will be based.

## Methods

### Design

To address the subjects’ experiences from having attended NPMP treatment, a qualitative interview was employed. This method is particularly useful when the aim is an in depth understanding of experiences from a first-person perspective. It allows for nuanced accounts of relevant themes and for exploring the meaning of the experiences for the subject.

### Participants

To achieve depth and nuance within the material, it was important that the sample of interviewees shared particular criteria related to the research question. The following inclusion criteria were applied for participation in the study: (a) subjects had a diagnosis of AN, (b) were at least 18 years old, and (c) were presently attending, or had recently attended, NPMP treatment of minimum six months duration. In addition, the therapist had to be an experienced NPMP specialist (i.e., minimum three years clinical practice). It may be noted that individuals with AN often have accompanying psychological symptoms, such as anxiety, depression, obsessive-compulsiveness and post-traumatic stress [43], all of which are likely to be reflected in the body and which inevitably will influence the findings of the body assessments as well as the approach used in treatment.

We had intended to recruit three to four participants. However, due to only a few of the specialist physiotherapists that we contacted having patients with AN in treatment, and due also to the pandemic (from February 2020 onwards) we only managed to recruit two participants who met the inclusion criteria. Both subjects were in their forties, were asked to take part in the study by their physiotherapists, and had been receiving NPMP treatment for more than two years. Despite the small sample, it was considered that the accounts from these two participants provided a rich and nuanced basis to illuminate the research question.

### The interview

A semi structured interview and an interview schedule consisting of the main themes of interest were used (see appendix 1). The interview procedure was flexible and allowed for following up on interesting points emerging during the interview and for moving back and forth between the different themes. Importantly, all themes were followed up by prompts like: “could you tell me more about that?”, and “could you explain that further, or give some examples?” The interviews were conducted, audiotaped and transcribed verbatim by the first author during the winter of 2019/20 and lasted between fifty and seventy minutes.

### Data analysis

Thematic analysis was used to analyse, interpret, and identify themes within the data. The analysis closely adhered to the six-stage process described by Braun and Clarke [44]. In the first phase, we familiarized ourselves with the material by reading and re-reading the transcripts and taking initial notes. In the second phase, we identified and coded interesting features of potential relevance to the research question. In this process, we looked for features at a semantic (explicit) and/or latent (implicit meanings) level, as this allowed for moving beyond what was explicitly said [44]. In phases three and four, the codes were organized into potential themes, the themes were reviewed to check whether they worked in relation to the coded extracts and entire data set, and a thematic overview was produced in which similar themes were clustered. In phase five, we started refining and naming the main themes and subthemes, and in phase six, the essence captured in each theme was summarised and the report was produced.

### Issues of quality and validity

In demonstrating scientific rigour and trustworthiness, Yardley´s criteria for assessing validity and quality in qualitative psychology are useful for ensuring high quality of qualitative research [45]. The criteria involve sensitivity to context, commitment and rigour, coherence and transparency, and impact and importance. It is recommended that sufficient quotations from the data are used to illustrate the points made and to show density for each theme (e.g., for N = 1–3, quotations are needed from every participant for each theme; for N = 4–8, quotations are needed for at least three participants per theme) [46]. The present study adheres to Yardley and Smith’s guidelines with regard to scientific rigour and trustworthiness.

### Reflexivity

Reflexivity involves the need for the researchers to recognize that they always bring their own horizons of experience and fore-conceptions to the research process, and that these may have some effect on all phases of the research project [47]. The first author holds a Master’s degree in health care, with specialization in NPMP. She works in the clinic with patients presenting with various sorts of bodily constrictions, including individuals with eating disorders. The second author has a PhD in sport sciences and works in academia. She is an experienced specialist in NPMP and has worked with individuals with diverse issues, including AN. We are aware that our previous experiences and understandings may hinder the process of allowing the new to speak in its own voice, but also that our past experiences might represent a way in to the new, as they may allow us to empathize and understand the participants’ voices and help them explore in depth the meaning of significant experiences.

### Ethical concerns

The study was approved by the Norwegian Centre for Research Data (NSD) in September 2019. The participants were given written information regarding the research project and were informed of the possibility of withdrawing from the study at any time. Both subjects gave written consent to take part in the interviews, and for the material to be analysed and anonymised before being published. To protect participants’ anonymity and safeguard confidentiality, names and identifying features have been altered throughout the analysis and presentation of the material.

Since the interviews dealt with themes which could potentially trigger difficult feelings, the subjects were encouraged to take breaks, if needed, during the interview and to contact the interviewer if they needed to follow up on any issues after the interview. One of the subjects asked for a short break during the interview, while neither of them made contact afterwards. In addition, we presumed that both subjects had the opportunity to process and reflect on matters with their present psychomotor physiotherapist.

### The attended treatment: Norwegian psychomotor physiotherapy (NPMP)

NPMP is a method developed in the Scandinavian countries since the Second World War, and is a much appreciated approach often used for patients with AN in these countries. The method is grounded in the understanding that lived experiences and feelings are embedded in and expressed through the body and that the body is a functionally integrated entity in which constriction in one part of the body influences the entire body [48]. The key idea is that body and psyche are integrated, and that unresolved and problematic feelings are contained in and reflected in the body. This involves the autonomic nervous system, which relays experiences of stress to the respiratory system, typically resulting in tension in the muscular system and constricted breathing [49] The method is considered valuable for addressing bodily constriction and disintegration in subjects with a history of high levels of life stress.

The approach is not specifically aimed at treating particular diagnoses or symptoms. Rather, by the use of massage, grounding and balancing exercises, it aims to achieve a general readjustment of bodily restriction, and to increase bodily flexibility and stability, in addition to enhancing connectedness to and awareness of the body and integrating the body in the self. The therapy includes facilitation of changes through careful release of bodily tension and constrained breathing in close collaboration with the patient. It has been shown that NPMP-based interventions help patients tune into their bodily sensations and feelings [50, 51]. The therapeutic process usually continues for some time (several months to years), as the individual needs to integrate potential changes and their implications at their own pace. They also need to comprehend what the various bodily constraints and rigidities may convey or communicate.

The treatment is based on a comphrehensive history taking and a thorough body examination typically involving assessment of four main dimensions: posture, respiration, muscle tension and texture. Functioning, including flexibility and ability to relax, is also considered. The intention is not to search for detailed impairments caused by medical conditions or injuries, but to consider how findings influence the functionality of the whole body and indeed the individual subject. While the results of the interviewees’ body examinations are not available in the present study, a number of bodily divergencies within the four dimensions have been described in other relevant work [16]. Kolnes has detailed findings from NPMP-based examinations of six women with AN who took part in interviews about excessive exercising. She found substantially restricted respiration (i.e., reduced inspiratory depth and thoracic stiffness), postural de-alignments (including forward head posture, protracted and elevated shoulders), increased tension in muscles in multiple parts of the body, in particular in muscles associated with the function of breathing, stiff standing position and reduced ability to relax, all of which affect the functionality and stability of the whole body [16]. In line with the above assertions on embodiment, it can be anticipated that a constrained body is crucial to structuring all experiences of the self and relationships with the world [52].

Taking into consideration the dynamic interaction between breathing and psychological states [53–55], the observing of respiration and changes in its patterns, rhythm and depth, is key to NPMP, and distinguishes the method from traditional physical therapy. Constrained breathing and increased muscular tension – dimensions often identified in subjects with AN during thorough physical therapy examination [16] – are held to be associated with avoiding or suppressing problematic feelings (e.g., fear and anxiety). A continuing pattern of restricted breathing does not merely affect bodily expressions, functions and movements [49], it also adversely affects mental awareness [50]. When the breathing is normalized and diaphragmatic, changes in posture and movement patterns are likely to occur spontanously, often along with an enhanced ability to relate to one´s feelings and to articulate difficult feelings [50].

## Results

Three main themes were identified in the analysed material relating to the subjects’ experiences with psychomotor physiotherapy: *the meaning of the therapeutic relationship, changes in bodily connectedness and ways of moving*, and *improved ability to articulate and reflect on feelings.* Verbatim citations from the informants are used to support the analysis.^1^

### The meaning of the therapeutic relationship

This theme comprises qualities of the therapeutic relationship pointed out by the subjects as essential qualities of the treatment. Feelings of being seen and taken seriously were features helping the subjects feel in safe hands during the treatment. Predictability and clear communication about what was going to happen during the treatment session, were also emphasized. Working with therapists who were understanding and who adjusted the treatment according to the situation and personal needs of the subject provided confidence and reassurance, as illustrated by Nora:

*I met a psychomotor therapist who clearly signalled that we’re going to do this on your terms. She said ´we’re going to help you, and for that to be possible we need to make sure that you feel safe in this situation`.*

Being able to influence where in the room the therapist should stand was highlighted as important by Frida when she lay on the treatment bench, as this meant she always knew where the therapist was positioned and she could see the therapist throughout the session. Having the power to influence the therapist’s position in the room helped her feel secure, it allowed her to feel prepared for what was going to happen:

*She always lets me know in advance what she’s going to do. (…) I like her standing behind me, or to the side, so that I have a sense of being in control. I like to have the wall behind me rather than a door, for example. It’s a bit like I’m able to decide her position in the room.*

Frida´s preference for having a wall behind her head when lying on her back on the couch, instead of a door, reassured her that nobody could enter the room from behind, and from an angle she could not see.

Having their personal boundaries respected and being advised by the therapist as to which part of the body the therapist would be touching felt important to both subjects. For example, at times Frida had felt that she ‘disappeared’ from the treatment situation for a while if something was uncomfortable or difficult. In such situations, it was crucial that the therapist was aware of this and followed up on it. The therapist might ask what happened just then and where had she disappeared off to. This enabled her to verbalise what she was thinking and feeling, which made her feel seen and looked after. Likewise, Nora pointed out that being asked questions like ‘*how is this for you, that I’m touching you here’, or ‘is it okay for me to touch you here*’, was important for her to feel respected. For her, such touching could otherwise be challenging, and the stomach was a particularly difficult area. She therefore felt it was important that sufficient time was set aside for the session, so that the therapist could approach her body in a careful and gradual process, thereby enabling her to become more comfortable with the treatment situation.

Gradually, as the subjects found the treatment situation to be safe, it felt easier to allow themselves to be challenged with respect to their own boundaries. This took both subjects a long time to achieve, but as their relationship with the therapist developed, a sense of safety was engendered that made it easier to challenge their own boundaries. For example, being able to remain fully dressed throughout the session helped Nora let go and remain focused on what was happening in the treatment room:

*There’s a lot of shame that comes with having an eating disorder, it’s a bit self-inflicted as it were, and not very easy to control. You can go there (to physiotherapy), and there is no need to perform well, or anything. (…) You are there because you’re in a mess and things are difficult, not because your shoulder is hurting. (…) I remember it felt so good not having to get undressed, I could actually receive treatment with all my clothes on. For me, with my anxiety about my body, that was great. (…) I noticed that calmed things down a lot.*

The quotes indicates that Nora found it easier to cope with the illness and with herself when she felt accepted by the therapist. She felt that the therapist was open-minded and understanding, and she did not feel judged in any way. A slow pace of treatment progression and being able to influence the frequency of sessions, offered predictability and were of great importance to both subjects. For Frida too, it took a long time to feel safe. Occasionally attending a double treatment session was therefore important in order for her to become relaxed during treatment, as this was more challenging in the first phase of the treatment period.

Furthermore, an important aspect connected to feeling confident and well protected during the treatment session was the experience of being ‘tucked up’ in a comfortable blanket by the therapist. Here Nora spoke about how she felt this added to her feelings of being safe and how it provided a feeling of being framed within the confines of the bench:

*At the end of each treatment session, the therapist pressed her hands firmly against my head and the rest of my body all the way down to my feet. This made me feel as if I was ‘enveloped’. I was allowed to just lie there and relax. It was really nice, for that meant you were lying contained within those confines. (…) What I’m left with, is this enormously good feeling of being safely tucked up.*

To be tucked up and contained within the confines of the bench and blanket worked well for her, particularly when she felt down. It allowed her a sense of ‘not oozing out’ on the couch. She declared that she could sometimes feel that her body was enormous, but here, when she could lie ‘contained within those confines’ and relax, she felt good as well as calm. For Nora, this was one of the aspects of the physiotherapy treatment that she felt she benefitted greatly from.

Moreover, feeling safe during the treatment provided the women with the opportunity to reflect on demanding situations in their everyday lives. The psychomotor physiotherapist could take the role of a mentor and sparring partner in relation to reflecting on important life situations. To be able to reflect on such issues, in general, it seems essential to feel safe in one’s surroundings and with the person you are talking to. This was indeed the case for the informants vis-à-vis their physiotherapists. Nora was able to raise diverse issues that concerned her, and felt that her conversations with the therapist helped her regain a more natural relationship with the social world. She could be looking to the physiotherapist for confirmation of what was to be considered “normal”, and she found that it became easier to seek normality the further she progressed with her treatment.

### Changes in bodily connectedness and ways of moving

*When you´re living with an eating disorder you tense up everything in your body, … you´re likely to hold in your stomach and raise your shoulders and you get a lot of tensions in your body. You´re really very focused on one thing. So I used to walk around with constant pain in my body – I was extremely tensed up.*

From being extremely tensed up in their bodies, as Nora vividly puts it in the above quote, the women experienced a number of changes in the experience of the body in the course of the treatment. Feeling more connected with the body and new movement experiences are concepts encompassing these changes. It appears that it became more natural, for both subjects, to be alert to their own bodily signals and to feel connected with their own bodies. They also felt the treatment facilitated awareness of how various structures of the body were connected, how changes in one body part involved changes in other parts, and added to their experience of the body as a whole in keeping them centred.

This point may be illustrated by feelings of being more grounded when, for example, sitting in a chair, which is the concept used by Frida when she speaks about how she better could sit *in* the chair rather than *on* the chair. She uses `grounding´ to indicate how she could actually sit down and take support from the chair, as opposed to perching on the edge and not being supported by the whole seat and back of the chair. She explained that having an eating disorder often means that many thoughts are racing through her head, but that this changed after the grounding exercise in the chair, which typically started off each treatment session, and during which the therapist helped her work her way through her body, part by part, allowing her to sense where she tensed up so that this could be followed by relaxation. By such grounding she became more able to sit still and more able to let go of her racing thoughts and it also boosted a sense of being present, mentally as well as bodily.

Moreover, Frida increasingly noticed that she could revisit this sensation in her everyday life. She found herself sitting down properly in other daily situations, rather than on the edge of her seat as if she was about to head off somewhere. By sitting comfortably with her body supported in the chair, she found it simpler to just sit and do nothing. She had, for example, noticed that:

*Recently, I´ve been flying quite a bit, and I´ve recognized that I now actually sit down in the seat on the flight. … It is a place you can´t do much else, but you can actually be sitting there, without fiddling with your mobile, or holding onto a magazine, and just be there.*

Merged with feelings of being tensed up in the body, are difficulties associated with the breathing and in particular with problems changing the pattern of breathing, since the subjects’ dominant breathing had typically been high costal for years, as illustrated by Nora:

*I´ve had a lot of pain in my stomach, there´s been a lot of pain, like in the upper chest, and the like. You see, my breathing has been constrained for a long time, I´ve had a lot of headache and shoulder pain, right. I´ve felt very tensed up in my body and felt constantly controlled, so that my body has been extremely, yes, extremely tense, as if it has been in a crisis state of readiness, all the time.*

The quote illustrates how constrained breathing may appear in combination with pain and tension in the body as a whole. Since breathing, and in particular diaphragmatic breathing, is a key focus of Norwegian Psychomotor Physiotherapy, the women spoke of experiences in which their breathing was eased. But they could also feel that it was challenging to address their breathing, as explained by Frida:

*She (i.e. the therapist) is very good at asking ‘what’s happening to your breathing now, what do you notice?’(…). And then I have tried to relax my tummy … I struggle with that, I haven’t really been able to do it yet. So my breathing isn’t quite there, it’s difficult…*.

Nora reported that she had become better at taking the time to breathe, and at reminding herself to do so in her everyday life by, for instance, setting aside time before going to bed to focus on her breathing. At one point she had resumed a sport which she used to greatly enjoy as a child. Taking part in activities she used to enjoy in the past helped her loosen up tensions in her body. It provided her with a sense of moving and breathing more freely, and also with a ‘time-out’ from everything that was painful and difficult, at least for a while.

Frida further described new ways of moving, and how the treatment helped her become more aware of her posture and how she uses her body. She explained that the new movements feel more natural, and that she now walks and sits more freely. While she previously had little contact with or awareness of muscles keeping her in the upright position, she has now become able to use them more purposefully in everyday life situations. For example, her awareness of how she walks and walking as such has improved:

*For example, pushing your heels down, and your toes, the whole foot. You’re meant to feel the whole outline of your foot. (…) I have always tripped over a lot, I’ll trip up even if there’s just a tiny stone … but I’ve noticed that this is changing, because I tend to lift my feet or my legs in a different way to how I used to. And I´ve been trying to walk up steps and to walk on stony ground and such, to make myself more conscious of the fact that you actually have to lift your whole foot, not only engage your hip. Thanks to this I now have less pain.*

At the beginning of the treatment period, Frida said she was walking as if she was marching, because she had the idea that she had to lift her legs properly. She now feels that she walks more freely and less statically, that her gait has acquired a better rhythm, and that she also has a better control of movements in general. In addition, she has discovered how her new way of walking has had a positive effect on aching and tension in other parts of the body, such as the neck.

Nora reported similar experiences. She found that her pattern of movement changed when she learned to take the time to do the exercises properly. For example, spending more time on.

moving more slowly from forward-leaning seated position to an upright position, and being more present in the movement, helped her experience elementary and straightforward movements in a new way. She declared that it was, however, important that the therapist provided her with exercises that were tailored specifically to her, which were easy to do, and which she could integrate into her daily routines.

Reduced physical unrest, and feeling less driven to exaggerate training activities, were other effects of the physiotherapy treatment reported by Frida. She better understood the adverse effects related to her previous physical activity level, and had become more conscious of the need to take breaks in her everyday life, rather than pushing herself in relation to physical activity. It has become easier for her to sit at ease and listen to her body´s signals:

*I am a bit focused on control, you know (…) Before, if my body was aching … Rather than taking it easy, I would walk an extra three kilometres, … instead of taking account of the fact that ´you should actually know that you really can’t go for a walk just now, you need to find something else to do. But no, I’m all set to go out, so I hobble along (…).´ I can now say that today, no, today I won’t be going for a walk (…) I still find it difficult, but I’m now able to not go for a walk.*

Letting go of some of her control meant that she now felt less concerned with exercising compulsively. Frida said that with hindsight she had realised that living as she had done before was exhausting. Reflecting on her choices had made her feel better about letting go of some of her obsessive rituals associated with excessive activity.

### Improved ability to articulate and reflect on feelings

Changes in relation to acknowledging one’s own feelings is the third key theme identified through the analysis, and involves features associated with increased ability to put feelings into words and differentiate between emotional nuances. In addition, the analysis revealed how these aspects might have influenced the way the subjects relate to others and made them more capable in social and relational settings.

Both subjects stated that they previously had had difficulties in articulating their own feelings, and that this was also the case during the first phase of treatment. During the course of treatment they felt they became more attuned to their feelings and found it easier to accept and take ownership of them. As the treatment progressed, Frida said she became increasingly aware that she had a tendency not to accept her own feelings, and that she even had a tendency to deny them. For example, instead of saying ‘*I feel*’ Frida would previously externalise her feelings and say ‘*people feel*’ and ‘*people recognise*’, when actually talking about her own feelings. The physiotherapist addressed this during the treatment and would stop her when she spoke about herself without using the first person. This helped her become more aware of how she expressed herself when talking about herself and her own feelings:

*We’ve practised this, it’s about speaking about myself as myself (…) Just the way I talk about other matters. I could also say ‘people talk about’. Somehow that’s much easier (…) So I correct myself when I’m speaking (…) It takes time, I must practise, but I feel it’s improved. Now we’re talking about things that actually apply to me and not to someone else.*

Through the dialogue and reflections with the therapist, she became more aware of picking up on how she really felt, and better at expressing her state of mind in words. She found that she was better able to put her feelings into words and say clearly when something was difficult. For example, now she can say: ‘*No, now I’m upset*,’ in situations where she earlier would have said: ‘*It’s fine*,’ or ‘*This is good*.’ To articulate her own feelings remains, however, difficult, but Frida feels she has made some progress in the right direction.

Nora described similar experiences. When she started with psychomotor therapy, it was inconceivable for her to show her feelings, either during treatment or elsewhere. She said she had always tried to be a ‘clever girl’, maintain a facade and fix everything herself, and above all, not show her feelings. She described how a storm could be raging inside her, while on the outside everything appeared to be in order. As she developed a feeling of confidence in relation to the physiotherapist, it gradually became easier to drop the facade and to allow her feelings to surface. She found that the dialogue with the therapist became more important as treatment proceeded, and because of the opportunity to broach different topics with the therapist, she felt she gradually gained better contact with her feelings and could more easily accept them. For example, after a difficult meeting at work, she could reflect on her own feelings like this:

*So, there’s a situation I find very difficult. So I take it up with someone and ask them what they think of my solution, right. Instead of feeling like a failure and stopping eating, you see. I’m sort of exaggerating, but it’s really just more about processing your feelings instead of doing something self-destructive.*

Through reflection on such situations in therapy, Nora felt she acquired new perspectives and became better at standing up for her own opinions and stated: ‘*What other people think doesn’t really matter all that much.’* In addition, when she got into difficult situations with other people, she noticed that she expressed herself more clearly in the conversation and could clear up any misunderstandings more easily:

*If I find myself in situations that I feel are weird, I say, ‘But what do you mean exactly?’ or ‘What do you really think, where are we now?’ and ‘Why are we in this situation?’ It’s really strange but people react very well to this.*

Nora felt she was met with a positive attitude when she brought up things that could be difficult or unclear. She felt she had learned to express herself more clearly in therapy and could use the same strategy elsewhere in life as well. When the physiotherapist encouraged Frida to verbalise and elaborate on *her* thoughts and concerns, she found it easier to reflect on both having an eating disorder as well as on what was going on in the treatment. In that way, she felt that someone continuously motivated her to reflect on her choices, without it representing a big challenge:

*It’s the same as when I was talking about becoming aware of posture and the body and all that. There’s someone prodding you the whole time … without being too challenging. Because then it’s easy to get stuck. (…) But if there’s someone kind of prodding you the whole time, it can result in self-reflection.*

With this quote, Frida illustrates how her confidence in the therapist helped her navigate thoughts and feelings, as well as articulate these better. At the same time as she achieved a more nuanced relationship with her body and increased awareness of her own feelings, she gradually became more confident in herself and found it easier to participate more actively in social situations such as voluntary work where she would feel more comfortable and less tense than in the past.

## Discussion

This research investigated experiences of the body and self in subjects who had attended sessions of a specialized physiotherapy approach that aims to release bodily tension and restricted breathing to help subjects tune into the body and into how difficult experiences, relationships and feelings are also bodily. The findings demonstrate that the subjects were exposed to novel ways of experiencing and sensing the body and bodily states following the treatment. While the therapy represented a situation that was challenging, given its involvement of and direct focus on bodily recognition and awareness, it granted the subjects a place where they felt acknowledged, accepted and safe. As opposed to the subjects feeling disorganized, closed up and tense within the body, the therapists’ careful and non-judgemental approach helped create an opportunity for them to become engaged in the therapy, to be ‘tucked up’ and contained within the confines of the couch and the situation as a whole. In addition, the therapeutic situation provided a space for dialogue about what was going on in the session, as well as for reflections about ways of experiencing and perceiving the body, the self and their interactions with the surrounding world. To feel tucked up ‘within the confines of the couch’ might have helped moderate discontentment with body size, and feelings of ‘oozing out’, feelings that are linked to clients with AN attending physiotherapy [39]. Another dimension might also be that there is a sense of safety afforded by being ‘tucked in’ that can be soothing on both the psychological and physical level, and that their racing thoughts and anxious feelings slow down as a result of achieving greater bodily tranquillity.

The interaction with the therapist and the feeling of being fully acknowledged, was crucial for the subjects to give in and open up for the therapy. This is in line with previous studies describing how therapeutic receptiveness and sensitivity are key features for a meaningful therapeutic relationship between physiotherapist and client to occur [56–58]. Our findings confirm in particular results from a previous study exploring the meaning of the first encounter with the NPMP therapist in former patients with musculoskeletal problems [57]. Ekerholt and Bergland found that for the body examination and treatment to be helpful, the clients need to feel accepted and acknowledged, the therapist must show sensitivity towards the client’s boundaries and what is going on during the session should be predictable and clearly communicated [57]. The concept of acknowledgement may signify how we humans understand ourselves in light of others’ reactions and behaviours. In order to acknowledge yourself, you need to feel acknowledged by others; such self-respect develops through being met with respect. Acknowledgment is about being able to listen to the patient and take on board what is being communicated, while at the same time focusing on what is happening with the patient. Similarly, the notion of validation, defined as “being awake to, accurately reflecting, and conveying acceptance of a patient´s behavior, thoughts, or feelings” ([59], p 467) is described as a key contributor to therapeutic alliances across treatment modalities (e.g., compassion focused therapy, cognitive behavioural therapy and dialectical based therapy). Validation may capture the essentials of the physiotherapeutic interaction in this study and the ability of the therapist to tap into the subjects’ feelings and experiences [60]. Experiencing care providers as collaborative and validating has been linked to higher rates of treatment acceptability and retention, lower drop-out rates, and better symptom improvement in individuals with an eating disorder [61, 62].

How we as humans are met by others, and our need to feel acknowledged and understood, are important features in the development of the self. According to the American child psychiatrist Daniel Stern, a comforting, accepting touch and interpretation of a child’s bodily expression can confirm and develop the feeling of being understood [63]. Even though Stern focused on the infant’s development, parallels might be drawn to people at other developmental stages who are fragile in their sense of selves. What helps to create and build good, intimate relationships are the special moments when we share common experiences and/or emotional perceptions [64]. Thus, the informants in this study felt cared for and accepted as they were by the therapist. They felt their experiences were acknowledged and validated, and they were allowed to bring in their own voice, all of which was fundamental for them to unlock the body as a previously closed system and to integrate the body in the self [17].

From being bodily closed up with constricted breathing, muscle tension, inflexible and stiff movements, the subjects described experiences in which they managed, at their own pace, to perceive and recognize bodily sensations and signals more clearly. Through the therapists’ careful releasing of muscular tension, enabling of bodily stability and facilitating of a deeper and more spontaneous breathing, they managed to breath more freely, unleash and let tensions go, and become grounded within their bodies. The subjects felt they were able to act and move more freely, measures that were transferred into everyday life situations, including daily practices like sitting and walking, which although seemingly simple are not necessarily straightforward for individuals who are bodily constricted. By allowing themselves to sit down properly and be supported by the seat and the back of the chair, the subjects gained a sense of awareness, of being present in the here-and-now, and achieved a more relaxed way of sitting as opposed to their old habit of perching on the edge of the chair, as if constantly on the way somewhere.

These notions resonate with previous studies and theory exploring physical therapy and body awareness therapies within the field of mental healthcare, including for AN [16, 39, 65–67]. Traditional physiotherapy methods aimed at increasing body awareness and helping subjects with AN become more comfortable with and accepting of the body, involve various forms of massage, relaxation, movement and exercise [66]. Tailored physiotherapy interventions and other body-oriented therapies (e.g., relaxation, dance, tai chi) have been shown to boost bodily perception and pave the way for new movement patterns, greater body awareness and positive physical experiences in people with AN [56, 68]. Studies exploring the effects of body awareness therapy and embodiment in patients undergoing psychiatric treatment, demonstrate increased awareness of physical sensations, better knowledge of the self, and improved relational skills [65]. The meaning of `being in one´s body´ is closely related to how the body is balanced and the ability to stand firmly on the ground. A balanced body is in general associated with integration of proprioceptive and sensory information mediated through both movements and static acts such as sitting and standing and which may generate a more coherent experience of the body as well as of the self [16].

In accordance with previous studies and theoretical work, our results indicate that various forms of physiotherapy (i.e., NPMP and body awareness therapies) have the potential to help subjects with AN become attuned to their bodies and to sense the body from the first-person perspective, as well as to (re-)establish the body as the centre of experience and integrate the body in the self. From previously being bodily constricted in their interactions with the world, integrating the experience of the body in the self and living it as their own are assumed to play a key role in how people relate to and encounter other subjects in the social world [32, 35]. These features are in line with phenomenological ideas inferring that for the self to belong to the world, the body needs to incorporate a lived subjectivity [11, 32]; that is, the body is experienced as the subject of effort and of being under one´s own control, rather than being under the control of symptoms related to the illness (e.g., obsessiveness, compulsiveness). As Merleau-Ponty remarks, we only achieve this subjectivity by being a body and by entering the world through this body, “the subject that I am, understood concretely, is inseparable from this particular body and from this particular world” ([32], p 431). Such increased recognition of the body and self, and the sense of being situated in the world, may not merely boost the lived subjectivity and bodily being in the world, it may also offer new ways for individuals with AN to relate to and interact with others that may be valuable for managing their illness. In addition, a strengthened experience of the body may help subjects with AN feel more alive within the body, more satisfied and at peace with themselves, whereas poorly developed body awareness can impose a feeling of missing out on something important in life [65].

Along with experiences of improved bodily acceptance and recognition, feelings of being more situated within the body and a sense of bodily ownership, the subjects communicated increased confidence in themselves and improved acknowledge of their own feelings. They were better able to reflect on feelings, and to differentiate between and articulate feelings and their meanings more clearly. In becoming better acquainted with their own feelings, they also gained increased ability to accept their own feelings in relational and social situations. While subjects with AN seem to have a tendency to avoid negative feelings (i.e., anger, anxiety, shame or sadness) and resort to restrictive dietary routines, excessive exercising or avoidance of socializing in order to avoid addressing problematic feelings and conflicts with others [69, 70], it is possible that an improved ability to connect with and regulate feelings can help reduce the need to use such adverse strategies with others to regulate difficult feelings.

These notions concur with other work proposing that awareness of thoughts and feelings, along with increased capacity to identify, modulate, express and make sense of one´s feelings, are likely to progress when bodily recognition and connectedness are strengthened [65, 66]. Importantly, increased body recognition through being aware of the body from within, is considered crucial for patients to understand their own emotions and needs [65]. Within the realm of phenomenological thinking, one key dimension in the relatedness between bodily connectedness and feelings is that bodily experiences are associated with an awareness that the body is always present in the here and now, in all actions and movements [32]. Thus, advances in articulating feelings to family, friends and care providers may ease concerns of not being understood or acknowledged, or of being a burden, which habitually seem to occur in subjects with AN [61, 71]. Through therapeutic approaches heightening recognition of the body, self and feelings, associations might be shaped between what is going on outside and inside the individual, which in turn may create experiential possibilities to extend interpersonal relations and social life [39, 70]. Finally, it could be theorized that this capacity of connecting with and articulating feelings may help thoughts and feelings to become mentalized, and help bring “the non-mental into the realm of the mental” ([72], p. 105) in subjects with AN.

The particular significance of *breathing* in structuring the changes in the subjects’ experiences associated with awareness of bodily connectedness and feelings needs further clarification and could be understood from neuroscientific perspectives and viewpoints on breathing deriving from psychodynamic theory. It has long been contended that feelings and unresolved experiences are embedded in and interact with breathing and the muscular system [55, 73–77]^2^. The assumption that feelings are reflected in the body can be understood in light of neuroscientific perspectives on the effects of stress on the breathing. When the autonomic nervous system is triggered by psychological stress, a number of physiological responses are likely to occur, including in the respiratory system (e.g., rapid and/or shallow breathing). The regulation of the affected systems may become impaired [78] and specific breathing patterns can be generated when problematic feelings and thoughts are repressed over time, blocking both awareness and expression. This point is brilliantly articulated by one participant in Ekerholt and Bergland´s study in her reflection on what she felt had affected her constrained breathing before she had begun treatment:*“My thoughts had hurt too much, so they had been locked out – or rather, locked in. When anything unpleasant happened, it (…) affected my muscle tissue and breathing. It expressed itself in physical pain”* ([50], p 836). It appears possible that for many individuals with AN, constrained and upper-chest breathing is mediated by high levels of long-term life stress interacting with the AN condition and its implications. A continued state of constrained breathing and muscular tension will, however, have an effect on the experience and functioning of the entire body, including bodily awareness and the experience of the self, given that the self is inherently bodily [20].

Accordingly, when one is breathing more freely, tension in the muscular system is commonly released, and movements become more flexible and vital. In addition, subjects become more able to identify and make sense of such changes through conscious attention and verbal or non-verbal communication with the therapist, as was the case in our study. Our results are in line with previous studies describing how changes in patients’ breathing are associated with experiences of an enriched capacity for self-reflection, understanding and sensemaking of feelings and bodily tensions [15, 50]. By breathing “all the way down” into the stomach (i.e., diaphragmatic breathing)^3^, feeling more relaxed in the body came about along with feelings of being more mentally relaxed and more verbally capable of articulating feelings and meanings [50]. In addition, when breathing changes, changes in posture and movements are likely to occur spontaneously. While reduced tension in the muscular system seems vital to connect with the body and self, a change in the breathing appears to be *key* for subjects to become mentally present in the here and now, to open up for the `locked in´ feelings and to begin to accept their own feelings and put feelings into words. Thus, the “phenomenology of breathing” has a crucial role in structuring the experience of the body as well as the interaction with the social world in the subjects.

Our study has limitations. Due to the pandemic beginning in the spring of 2020, the same time as the interviews were due to be performed, it was difficult to recruit informants. It was therefore fortunate that two women with AN who had been attending NPMP sessions over a long period of time agreed to be interviewed. The study is based on data derived from these two informants, and the findings may therefore not be generalizable. Nevertheless, the qualitative interviews have provided rich descriptions of the participants’ experiences, with detail and nuance, from a first-person perspective. Their accounts and our analyses can provide insights into how physiotherapy may benefit individuals with AN by helping them to establish a more integrated experience of the body and the self. This paper is preliminary and explorative and needs to be extended with a larger sample. Research investigating subjective experiences of therapies addressing bodily awareness and the potential these may have to re-establish the body in the self, is limited, and such topics deserve more attention with respect to the treatment of eating disorders.

## Conclusion

Within the framework of phenomenology and embodied mind theory, this study provides a unique in-depth exploration of embodied feelings and distress in subjects with AN and their subjective experiences from attending a specialized physiotherapy intervention. The study illustrates how difficult experiences, thoughts and feelings over time may interact with and manifest in the body as withheld breathing and diverse bodily constraints that affect both self- and body awareness as well as the subjects’ social and relational life. The results indicate that the intervention had a number of benefits for the subjects in terms of enabling an integrated experience of the body in the self, an increased capacity to identify, express and make sense of feelings, as well as enriched possibilities to interact with the social world. They show too that it was crucial for the subjects to feel acknowledged and validated by the therapist throughout the process.

This study endorses the notion from phenomenology of including the first-person perspective when investigating the contribution of bodily experiences to the self and to our interactions with the world. It also illustrates the breadth of experiences and sensations of the body that may serve the role of `protecting´ subjects with AN against distress and difficult feelings, and which collectively may keep the subject from entering into a helpful process of recovery. Integrating the subjective experience of the body in a skilled and validating way is an important but often neglected field in treatment protocols for subjects with AN. Involving physio- and body awareness therapies that help patients relate to, understand and integrate bodily experiences in a validating manner may complement other treatment approaches in a beneficial way and help patients with AN proceed in the process of recovery. Lastly, the study may generate new insights into phenomenological conceptualisations of the experience of the body in general, and more specifically in terms of bodily constrictions, bodily experiences and their relatedness to the self in subjects with AN, which go beyond existing concepts and have the potential to extend existing models on changing eating behaviour, attitudes and cognitions related to body image concerns. Indeed, the results illustrate how our experiences of the body structure all our experiences of and with the world. This area clearly needs to be investigated further.

## Data Availability

Not applicable.

## Appendix 1

The interview schedule included the following themes:

- Why did you start NPMP treatment?
- Now that you´ve attended treatment for a number of months, could you tell me about your experience? (Prompts: How has it been for you in general? Have you experienced any changes? Do you sense any changes in your body? What about feelings?).
- Could you speak more about how you can sense changes in your body? (Prompts: If there have been any changes in your body, could you tell me more about them? For instance: How is your breathing? How do you move around? How do you stand, do you feel your feet? Any changes in your relationships and/or your social life?).
- Could you tell me more about what the changes mean for you?

## List of abbreviations

AN: Anorexia nervosa
NPMP: Norwegian Psychomotor Physiotherapy

## Footnotes

1 In the quotes, the informant’s pauses are indicated by three consecutive dots: ‘.…’. Omissions are indicated by an ellipsis in the form of three dots enclosed in brackets: ‘(…)’. Any text in brackets represents an explanation provided by the authors.

2 The theoretical framework of NPMP is influenced by Reich´s thinking about feelings being reflected in the body and how feelings interact with breathing and the muscular system. These ideas were further developed by the Norwegian psychiatrist T. Braatøy and physiotherapist A. Bulow Hansen.

3 Diaphragmatic breathing is considered the optimal breathing pattern. It implies that the diaphragm, which is one of the largest muscles in the body, descends when inhaling. When breathing movements take place in and elevate the upper chest, the movement of the diaphragm is reduced and an overuse of breathing muscles occurs, inducing fatigue and pain and reduced neuromuscular control of the entire body, especially the neck.
